# Fracture Characteristics and Energy Dissipation of Textile Bamboo Fiber Reinforced Polymer

**DOI:** 10.3390/polym13040634

**Published:** 2021-02-20

**Authors:** Chun-Wei Chang, Feng-Cheng Chang

**Affiliations:** School of Forestry and Resource Conservation, National Taiwan University, Taipei 10617, Taiwan; f08625002@ntu.edu.tw

**Keywords:** textile preforms, fiber reinforced polymer, fracture modes, fracture energy, bamboo fiber

## Abstract

The fracture theory of fiber-reinforced polymer (FRP) composites is complicated compared to that of homogeneous materials. Textile FRPs need to consider crimp, fiber off-axis and various weaving parameters in a two-dimensional scale, which makes research of failure and fracture difficult. The objective and main contribution of the present research lie in taking textile bamboo FRP as an example and using tools such as toughness, load and deflection curves analysis, energy analysis, and first-order derivative signals to establish the preliminary information needed for fracture theory. This is followed by observing the fracture characteristics of the material under bending. The identification of fracture modes, corresponding energy, and energy dissipation are all prerequisites for developing fracture models in the future. Differences in the direction of force, weaving method, and number of laminates will cause the amount and direction of fibers to vary, which makes the type and progression of fracture different. Combining signal analysis, fracture images and energy dissipation curves, there are different modes of fracture between various groups due to different energy storage forms and crack types, which ultimately lead to different energy dissipation behaviors.

## 1. Introduction

FRP (fiber-reinforced polymer) composites have been widely used in many fields of life. However, the current mainstream fibers are mostly made of inorganic or petrochemical raw materials, for example glass fiber and carbon fiber. In addition to the high cost of these fibers, other factors such as difficulty in recycling, energy consumption, irreversible waste, consumption of large amounts of chemicals, and high biological toxicity have caused many environmental impacts. Moreover, production disadvantages such as high energy consumption, difficult processing, and even various eco-regulations have also posed resistance to conventional fibers.

Natural fiber is widely used in daily life due to its accessibility, wide distribution and sufficient stock [1]. The biodegradable, anisotropic porosity of natural fiber products solves the problem of non-recyclability [2,3]. Natural fiber even provides further strength and lightweight characteristics that result in a high inducement. The surface roughness, aspect ratio, and flexibility of natural fibers are quite suitable for textiles [4,5]. The woven preform can reduce the manufacturing time during lamination and improve the processing accuracy. In addition, woven preforms can also provide better mechanical properties, impact resistance, damping and damage characteristics than those without weaving [6]. Natural fibers have great advantages as weaving FRP has become the mainstream nowadays. In the past decade, natural FRPs have become a popular research topic, especially in the fields of aerospace and engineering materials.

Today, it is imperative for natural fibers to return to the industry [7], however there are still many challenges in practical applications. For materials applications, the characteristics of fracture are related to the life and property safety for users. Predicting and preventing fracture of various scales has always been an important issue in engineering. The heterogeneity of FRPs, complexity of the woven preform, as well as unique behavior and variability of natural fibers make it difficult to grasp the fracture behavior. So far, there have been preliminary results in the failure theory of FRPs. The failure criteria for composite materials such as Hill-Tsai and Tsai-Wu can be used to make predictions for laminated composite materials. The earliest development of failure criteria used pure stress or strain to assess whether the external conditions exceed the maximum value that the material can withstand, and the well-known criteria are maximum stress and maximum strain. However, the anisotropy of FRP can lead to interactions between different stress components (such as shear stress or transverse normal stress). Therefore, the failure theories of Tsai-Wu, Hill-Tsai and Hashin were subsequently developed [8]. Nevertheless, these theories that treat each constituent material as a single phase cannot be applied to actual FRP fracture. This can be attributed to the fact that the composite has many different modes of fractures, which are affected by the direction, composition and external load conditions. The physical phenomenal-based criteria [9] were developed to evaluate corresponding failure criteria based on different fracture modes, and they are the most comprehensive prediction tool by far.

Due to the complex internal structure of composite materials, it is impossible to cover all failure modes by a single-phase behavior. Therefore, it is necessary to distinguish the types of failure (especially fractures). The fractures are divided into three modes according to differences between the stress direction and crack direction, namely opening mode (Mode I), sloping (Mode II) and tearing (Mode III). In fact, the mixed mode (Mix mode) is the practical situation. The linear-elastic fracture mechanism (LEFM) explains how cracks are generated and propagated under the microscopic view. There are many ways to test fracture toughness, most of which use pre-crack to simulate the stress of the crack tip. Owing to the special structure and anisotropy of FRPs, there are many ways to test specific modes. Some examples include the DCB test (double cantilever beam) that measures interlayer cracking (Mode I), the ESE (T) test (eccentrically loaded, single-edge-notch tension), and the SENB test (single-edge-notch bending). Although these tests can simulate the energy required for a single type of fracture, there is a significant insufficiency between the practical fracture behaviors. This is the reason why this research intends to use complete materials instead of standard specimens with prefabricated cracks.

None of the above theories simultaneously take into account the unique fracture mode, stress distribution and crack development behavior of natural fibers. Secondly, the above-mentioned failure criterion is used to predict conditions under which the material cannot maintain its original performance; it cannot reflect the different rupture modes. Although LEFM can be used to describe the conditions required for different fractures to occur, it is not sufficient to fully describe the composites behaviors. The occurrence of fracture is extremely complex; hence, it is difficult to accurately quantify and classify it in practice. The existing failure theories and behavior predictions are mostly based on tensile conditions [10]. While bending is also a common condition of loading, the analysis of fracture behavior under bending is rarely discussed. Discussion on the failure of textile FRP and its strain energy is still an incomplete field [11].

In this study, BTRP (bamboo textile-reinforced polymer) was made by using woven preforms composed of strips, which is a less commonly discussed type of bamboo fiber. Bamboo strips do not hinder performance due to sizing or twisting, and they can be made into any desired size and length through simple mechanical processing. Therefore, it is speculated that better performance in stress transfer, alignment accuracy and lamination can be achieved with low energy consumption and low dosage of chemicals. The woven fibers will have specific forms of undulation and arrangement because the fibers interlace each other. Therefore, the internal stress transfer is unique, and it also makes the fracture mode different. Before focusing on the destructive behavior discussed in this article, it is necessary to clearly define the fiber types. This research focuses on continuous, woven, non-twisted 2D textile FRPs. The crack propagation of woven FRP is completely different from that of non-woven FRP [12]. The crack direction and fracture modes will vary depending on the direction of the principal stress, indicating that the woven material has higher anisotropy [13]. The woven structure influences LEFM parameters in terms of areal density, fiber tow density, and fiber direction, etc. These are all related to the variables designed in the present work [14,15,16]. Another model closer to the scope of this study is the continuum damage model (CDM) [17]. However, in our study, the behavior of fractures (such as signal and load deflection curve) is divided by layer of preform. In addition, the main contribution of CDM is to describe and predict the crack propagation in a relative microscopic view. The fracture in our work is most intuitive and visible.

Based on previous studies, there is an important relationship between failure and fracture which is also affected by the textile structure. The existing research methods are divided into two different fields—discussing failure conditions at a macro-level and analyzing the required energy from a micro-perspective; they are both related to energy conversion. Regardless of the above methods, the establishment of the theory requires the consideration of complex fracture modes to be representative and practical. The main contribution of this research is the in-field observation of these fracture modes and their influences on energy dissipation.

Therefore, the objective of this study is to observe the destructive behavior under bending in two parts. The first part is to identify the modes of fracture that may occur in BTRP through the first-order derivative signal of the combination load-deflection curve, the fracture images, and the energy conversion. This includes fracture category, fracture signal density, the sequence of occurrence, rupture area and initial fracture energy. The second part discusses the impact of fracture characteristics on energy from the perspective of energy dissipation, and it attempts to summarize the key factors and mechanisms that affect these characteristics. The advantage of this method is that it needs neither to measure the properties of all components in advance nor to make a specific specimen. Using this method can directly reflect the behavior of specimens of all groups and conditions under practical loading conditions. In the future, the assistance of signals and images can be used to distinguish the energy required for the destruction of each layer. It is also possible to establish the sequences, characteristics, conditions, and even influences of various types of fracture through signal comparisons.

## 2. Materials & Methods

### 2.1. Manufacturing of Bamboo Strips and Composite Molding

**Figure 1:**
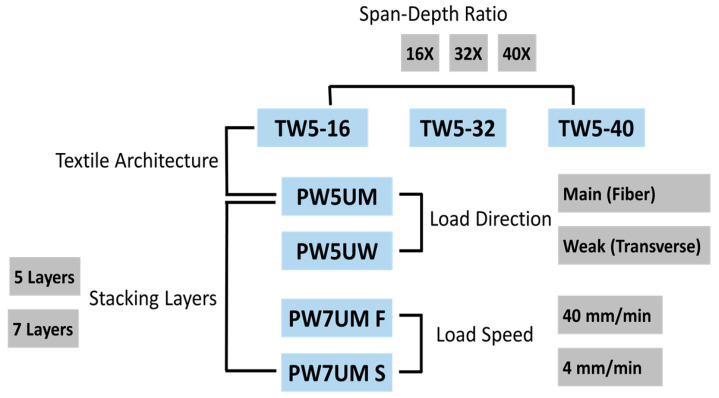
Specimens and test conditions of different factors.

In this study, bamboo strips made by *Phyllostachys makinoi* from Hsinchu, Taiwan were used as the reinforcing fiber of FRP. The bamboo strips in 0.5 mm (thickness) × 9 mm (width) were processed from solid bamboo directly with only mechanical slicing and kept the natural bamboo internal structure as a long and thin band-like form. Then, these strips were woven to form the lamina of the layered composite. The raw bamboo processing and bamboo strips were produced by local factories without any surface treatment. Epoxy resin for RTM SWANCOR 2511-A was used as matrix (Atech Comp co., Ltd., Kaohsiung, Taiwan), mixed with hardener at a weight of 10:3. The basic information of preforms and strips is listed in Table 1. The bamboo strips were processed into bamboo preforms by two weaving methods and then made into BTRP with different structures according to Figure 1. 

The factors included 3 structural variables (weaving method, load direction and number of layers) and 2 test condition variables (span-to-depth ratio and load speed). The weaving method was divided into plain weaving (PW) and twill weaving (TW). The different weaving methods would make fiber distribution, fluctuation and tow density different, in addition to affecting the mechanical properties. The number of layers would also cause different deformability under bending, and this research compared BTRPs with 5 layers and 7 layers. Fiber is the most important component of the FRPs in terms of mechanical performance, and the significant anisotropy of the fiber would affect the performance as well. The PW5U materials with two fiber directions were used to make flexural specimens with different load directions, and M and W represented the main and weak load axis, respectively. 

A 50 cm wide and 3 mm thick silicon bag was used as a mold to fabricate BTRP batch with VA-RTM. After the infusion was completed, it was naturally cured at room temperature for 6 h, and then placed in an oven at 80 °C for 8 h, before being cooled in an ambient environment to complete the post-curing process.

### 2.2. Quasit-Static Flexural Test

The bending test refers to ASTM D790 for a three-point load test, which was completed by an MTS Model 43 strength testing machine (MTS, Inc., Massachusetts, MA, USA) with a 2.5 kN load cell. The plane size of the test piece was 20 mm (width) × 300 mm (length), and the span was 32 times the measured thickness (note—the thickness of the finished product of VA-RTM varied with the number of layers and the weaving method). The flexural strength, deflection and bending elasticity were also calculated. Each group contained 3 specimens.

The two variables related to the condition in Figure 1 were used to simulate materials under different conditions of use. Polymer materials are sensitive to load speed, and their elastic behavior will significantly affect the material’s response to external energy. In this experiment, the PW7U specimens were loaded at 4 mm/min (S) and 40 mm/min (F), respectively. The last variable was the span-to-depth ratio. When the material is bent, there will be a horizontal shear effect (shear effect) accompanied by a bending moment, and the shear force will increase as the span-to-depth ratio becomes smaller. In laminated materials, the presence of shear can cause completely different fracture patterns. Therefore, in this study, three different span-to-depth ratios (16, 32 and 40) were designed for the TW5 specimens to simulate different shear forces. The rest of the groups were all 16. In order to fully inspect the fracture behavior of the material, the force and deflection signals were recorded from the beginning to the maximum load and until 5% of the maximum load was left.

### 2.3. 1st Derivative Fracture Signal

The complexity of the FRPs makes it difficult for the load signal to distinguish the occurrence of fracture with the naked eye. By obtaining the first derivative function of the force signal with respect to the displacement, and superimposing it with the instantaneous image sequence, the real crack behavior corresponding to the appearance of various strong and weak signals can be observed. With the actual fracture development in the image, the force signal positions of different modes of fracture can be separated. By integrating the curve of the load and deflection diagram with the deflection, one can calculate the energy required for failure and the total energy of the material.

### 2.4. Fracture Image Capturing

In this experiment, a CCD (charge-coupled device) lens (Largan Co., Ltd., Taichung, Taiwan) with a 12-megapixel and f/2.2 aperture was used to shoot the side of the material at 60 frames per second.

## 3. Results

### 3.1. Flexural Properties of BTRPs

Flexural properties such as modulus of elastic (MOE) and modulus of rupture (MOR) are the most basic quantitative descriptions. Table 2 shows the results of the ASTM D790 test. Fracture Toughness refers to the energy required for the primary failure of the material per unit thickness (that is, at the maximum load), which equals to the area under the curve of force and deflection. The total energy is the sum of the area under the complete curve. These properties are affected by variable factors and have similar trends to most woven FRPs, including the amount of fiber in the load direction, the number of laminas, and the weaving method [17,18]. Three of the structural variables listed in this study are related to the amount and structure of fibers. 

### 3.2. 1st Derivative Fracture Signal

**Figure 2:**
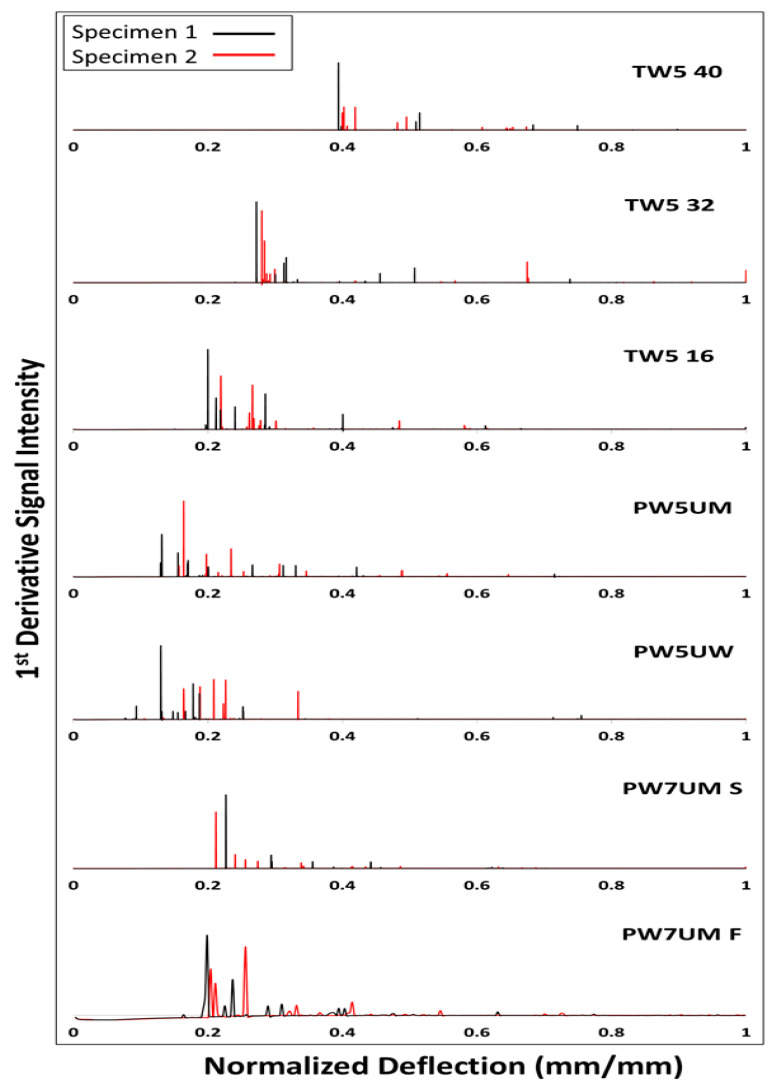
First derivative fracture signal of load-deflection curve with normalized deflection.

Figure 2 shows the signal diagram of the 1^st^ order derivative fracture signal. Signals from two representative specimens are selected in the scheme. The signal distribution range, relative intensity attenuation, deflection at fracture initial and intensity of each variable are not the same. It must be noted that the relative strength is only effective for a single specimen, which cannot be compared across test pieces or groups. These results could be used in further investigation such as numerical analysis like finite element methods.

### 3.3. Energy Dissipation Curve

**Figure 3:**
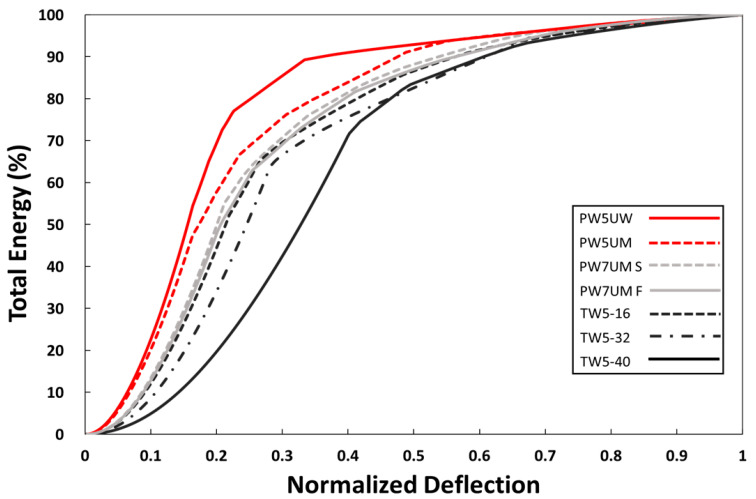
Normalized energy dissipation curve of BTRPs.

Through the cumulative integral curve of the load-deflection diagram, we can understand the behavior of the material in the process of material deformation and destruction, accumulating and releasing energy. After normalizing the deflection and energy, the energy dissipation curve in Figure 3 can be obtained. The lower curve distribution represents the energy dissipated more slowly; and the higher distribution represents the energy released by the fracture in the early stage of flexure.

## 4. Discussion

### 4.1. Effects of Load Direction

**Figure 4:**
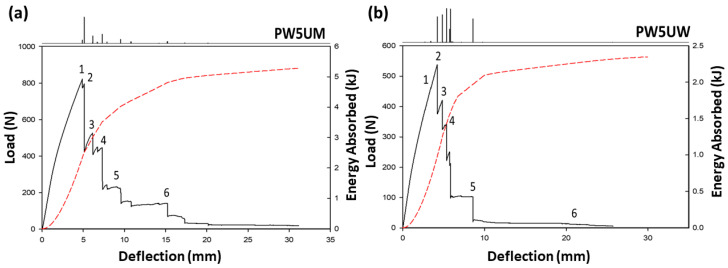
Load-deflection curve, accumulative energy curve and 1st derivative fracture signal. (**a**) PW5UM; (**b**) PW5UW. Numbers above the curve represent the corresponding fractography in Figure 5.

**Figure 5:**
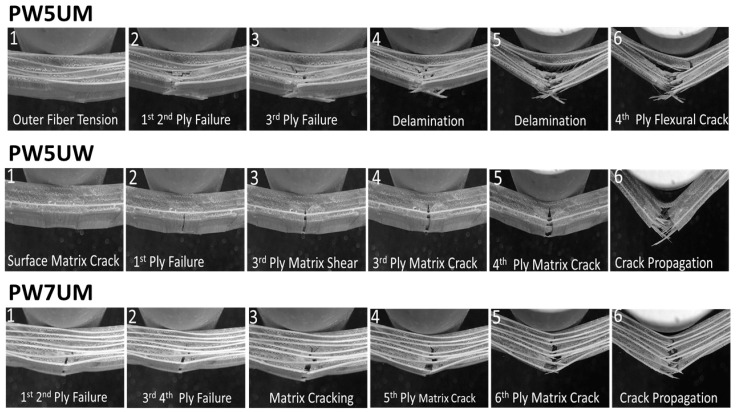
Fractography and fracture progress of PW5UM, PW5UW and PW7UMS specimens.

The comparison of load direction can be observed in the difference between PW5UM and PW5UW. The difference in quasi-static mechanics and fracture mode was the most obvious among the five variables. Figure 4 shows the load-deflection diagrams of the two groups. The upper panel is the signal of the first derivative function, and the red dashed line represents the accumulated energy. In the figure, it can be seen that PW5UW was pre-fractured due to the fact that there were fewer fibers in the load direction on the surface, resulting in a large deformation of the surface matrix, which generated a small signal before the main failure (Fracture 1 in Figure 4b). In addition, the intensity of the signal from 5UMPW gradually decreased, and the signal from PW5UW was relatively flat, with a more uniform spacing. Such differences were mainly due to the different modes of damage. The former fracture is shown in Figure 5, which was mostly a fiber tensile fracture on the tension side. The appearance was mostly characterized by a large area and instantaneous whole layer fracture. Higher total energy meant that a larger amount of energy must be consumed to damage the fiber. The latter damage was the matrix crack where the crack developed vertically, and the damaged area was small.

**Figure 6:**
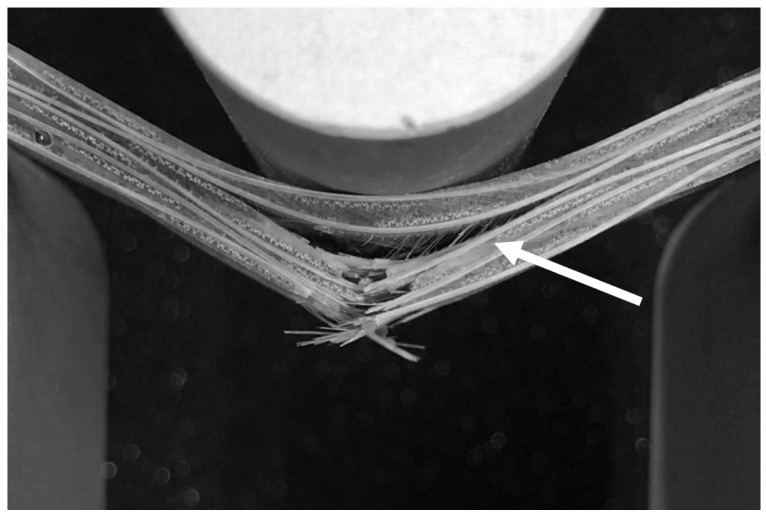
Fiber bridging of delamination obtained from the PW5UM specimen.

It can be seen more clearly in Figure 2 that PW5UM and PW5UW had different signal distribution ranges. The failure progress of PW5UM included other different fracture modes in Figure 5, such as instantaneous horizontal delamination (4), interlaminar cracking (5), and later turning into matrix flexural fracture (6). These characteristics are also reflected in the load-deflection curves. PW5UW had fewer axial fibers and limited load capacity. Therefore, the matrix of PW5UW broke directly and gradually developed upward, causing layer-wised cracking. Each peak on the load force curve was relatively similar. Finally, those with more axial fibers were also prone to fiber bridging effects (Figure 6), which made them resistant to more external energy. In summary, the fibers in the axial direction of the force were one of the important factors of fracture.

### 4.2. Effects of Preform Structure

In addition to fiber direction, the surface density and fibers volume fraction also made the failure characteristics different. PW5UM and TW5-16 were compared with the same force conditions, both being five-layer groups, in Figure 2 to understand the effect of the preform structure. It can be seen from Table 1 that there were more fibers per unit area in twill preform. Continuing the observation of the previous section, it can be inferred that TW5-16 should be more resistant to surface tension. This can be confirmed by the delayed occurrence of initial fracture signal. The destruction of fibers would be dragged by the cells, so it would show gradual and more complex rupture. The signals of TW5-16 were denser and coexistent with each other, and the total number of signals was more than that of PW5UM. The last point, different from the aforementioned initial failure, was that when only 1 to 2 layers remained, the stiffness of the PW single lamina was significantly lower than that of TW, and it could withstand greater deformation. Therefore, both PW5UM and PW5UW had a wider signal distribution range than TW. The higher MOE of TW5-16 than PW5UM in Table 2 can also confirm this argument.

### 4.3. Effects of Preform Layers

The difference between PW5UM and PW7UMS was only found in the number of stacked layers and the corresponding thickness of the FRP product. The fracture sequence of PW7UM is shown in Figure 5. The mode and characteristics of the fracture were similar to those of PW5UW. The fracture range was small, and the propagation of cracks was also concentrated and quickly developed to the upper layer. However, the arrangement direction of PW7UM was similar to that of PW5UM. In Figure 2, the PW7UMS signal appeared significantly less and small. In terms of the distribution range of the signal, it was also very different from PW5UM and PW5UW, which only differed in the number of layers.

This must be explained from another aspect. The results in Table 1 show that PW7UM had higher modulus and strength. MOE is related to the section modulus of the material; hence, thicker materials would have higher MOE given the effect of specimen geometry. However, it must be noted that only the VA-RTM or other processes that rely on atmospheric pressure for mold clamping would have the characteristic of “increasing thickness with the number of layers” [19]. Regardless of whether it affects thickness, in most studies, the higher the fiber content, the higher the modulus. However, the strength does not necessarily have a positive correlation with the number of layers and it may be affected by the size effect. In the VA-RTM process, it is also possible that with more layers, the relative fiber content would decrease due to the weaving structure, thus resulting in a decrease in strength [17]. However, there is no conclusion yet that this trend may be the opposite with the preform type [20]. In this test, the higher strength may suggest a higher fiber content or higher volume fraction.

The higher fiber content in the same preform structure means the distance between layers is closer. It also means that the pure resin matrix layer between the layers is thinner. Cracks in the FRP would be generated in the direction of the “easiest development”, that is, the least resistance. According to [21], fiber delamination is the most important mode of fracture after surface fiber tensile fracture. The key reason of delamination is mismatch between material phases. The fiber and matrix react differently to stretching, so that horizontal shear force is generated between the interfaces and eventually leads to failure. Due to the thicker resin layer, the mismatch of PW5UM was more significant than that of PW7UMS. Therefore, in the process of fracture for the first few layers, there was a large-scale delamination, and the crack would develop along the interlaminar surface. However, the interlayer state of PW7UMS was more difficult for cracks to develop between layers, and fiber fracture was the main mode. It is worth mentioning that although the rupture area was similar to that of PW5UW, the damage completely dominated by the matrix differed from that of PW7UMS. PW5UW was characterized by a flat crack perpendicular to fiber break, while PW7UM was a mixed failure of fiber and matrix. 

However, even from the strength and fracture type, it can be inferred that the difference between PW5UM and PW7UMS was due to higher fiber content. The scope of this research cannot prove that the fiber content was changed by the number of layers. Fiber volume fraction is one of the key factors. However, the accurate volume fraction is quite difficult to obtain due to the characteristics of VA-RTM and continuously changing thickness of specimens. The effect of the number of layers cannot be fully concluded in this study, and further research is still needed. 

Regardless of the quasi-static mechanics, energy characteristics, fracture signal or fracture image, it can be found that load speed had no effect on fracture behavior. Only in Figure 2**,** the signal was blurred due to the faster load, and there were no significant differences in the rest. 

### 4.4. Effects of Span-to-Depth Ratio

**Figure 7:**
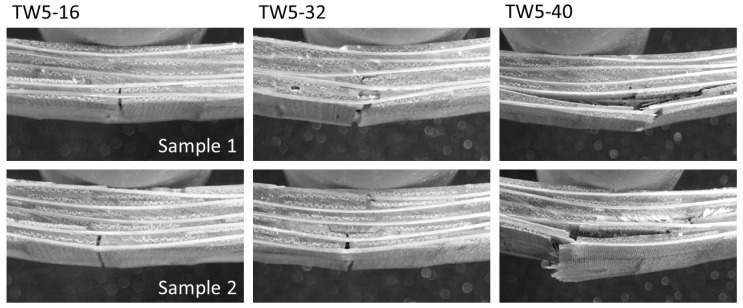
Main fracture of the TW5 specimen under different span conditions (corresponding to the first signal in Figure 2.).

**Figure 8:**
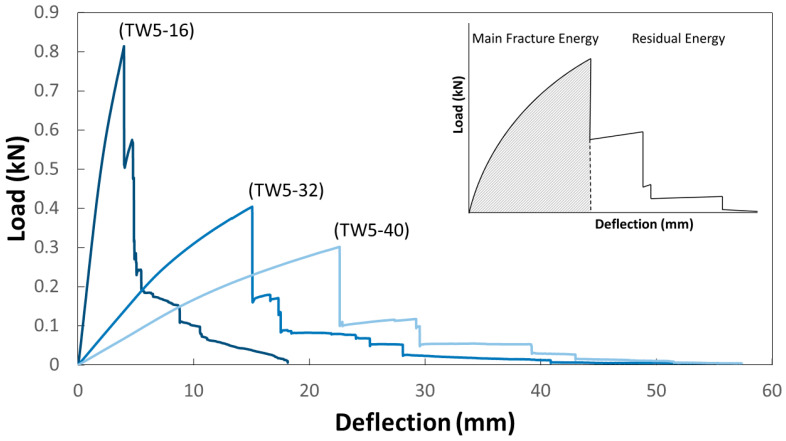
Load-deflection curves of the TW5-16, TW5-32 and TW5-40 specimens.

From the signal in Figure 2, the larger the span, the less the number of broken signals, and the initial was shifted. The large-span materials can withstand greater deformation. In addition, TW5-40 was characterized by a high-intensity primary fracture signal, followed by several small signals with a huge drop. The larger the span, the greater the attenuation between the initial signal strength and the subsequent signal. From the fracture images corresponding to the first signal of TW5 with different span-to-depth ratio (Figure 7), it can be inferred that the above difference was caused by the severity of the initial fracture. Comparing the fracture image corresponding to the first signal, TW5-40 had the largest fracture area, which may be distributed across three layers and accompanied by a huge inter-layer fracture. The largest span in Table 2 also had the highest fracture toughness, which represented more energy accumulated before failure for a fierce release. This inference can be further confirmed in Figure 8. The residual load capacity of TW5-40 after the initial failure was significantly less, with a load remaining of about 42%. The TW5-16 still had 69% performance after the primary fracture, showing that there were still fiber layers that could bear external load. The difference in the area under the curves before and after the primary fracture increased as the span became larger (with the span from small to large, 8.4%, 33.3% and 43.5%). It can be inferred that the energy of TW5-40 was mostly dissipated when the primary fracture occurred.

The mode of fracture was the opposite of the originally designed shear effect. The group with the least shear had the most serious shear fracture. This may also be related to geometric effects—when the span was small, the material did not deform easily, so the external energy of TW5-16 accumulated in the material body, which was reflected in the larger slope in Figure 8. TW5-40 was better able to withstand deformation, so the applied energy would be transformed into material displacement. It can also be inferred from the delay and slope of destruction in Figure 8 that the energy accumulated more slowly in the material body. However, when the ultimate deformation was reached, the cumulative total energy including deformation was higher than that of TW16, so the fracture after the initial release was more serious [22]. In the meantime, when the deflection was larger, the shear force between the layers would be larger, so the fracture was an obvious interlayer failure, as shown in Figure 7. The shear force conditions designed with the span-to-depth ratio would not be reflected in fracture behavior, but the most severe shear fracture would occur if BTRPs can withstand a larger amount of deformation. On the whole, the behaviors of the three groups of TWs were all related to energy dissipation and resistance. From the standardized energy dissipation curve (Figure 3), TW5-40 specimens could transform energy through material deformation. Therefore, the energy accumulation that increased with deflection was slower, and the curve was distributed in the lower right; TW5-32 and 16 were broken at lower deflection, so the curve was closer to the upper left. Through Figure 3, it is also possible to compare all variables and draw conclusions based on energy. Deformation will store energy and destruction will cause energy to dissipate.

The amount of axial-loaded fibers was less. The fibers cannot carry the energy, so they were destroyed earlier with the energy released. These led the PW5UW curve to be located above PW5UM. Compared with PW5UM, TW5-16 had more fibers in the plane, and it could also slow down the rapid release of energy. Based on this, the curve can be used to confirm that PW7UM may have a higher fiber content than the five-layer one, because of its lower curve distribution. The load speed was not related to the fracture behavior and it can be confirmed in two coincident curves. Combined with the comparison of the fracture images, the following conclusion can be made—the distribution of the curve was affected by two characteristics. 

For the curve of BTRPs on the upper left, the fracture behavior may be dominated by matrix. The lower the curve location, the fracture behavior was more likely to be dominated by fibers. The second characteristic was the material’s tolerance for deformation. The ability to store energy through deformation was characterized by a curve that shifted to the lower right, otherwise it would shift to the upper left.

## 5. Conclusions

This research analyzed the failure behavior of woven bamboo fiber composites under bending by combining image capture and derivative signals. The aim is to identify the modes of fracture and discuss the impact of fracture characteristics of energy and summarize the key factors and mechanisms. Results showed that the fracture signal, fracture image, and energy curve can distinguish the influence of different variables on fracture behavior and provide definite evidence for each other. The difference in the direction of load, the weaving method and the number of laminates caused the different number and distribution of the fibers, as well as different modes of fracture and development progress. The effects of test conditions were different from the original assumptions. The original plan to create different shear forces by adapting span-to-depth ratio was invalid, and the final fracture behavior and characteristics were contrary to the expectation. The relationship between the number of laminas and the fiber volume fraction still needs further investigation. Through the distribution of the energy curve and discussion, the following two conclusions can be drawn. First, the fracture is a path of dissipating applied energy. With different fiber structures, there will be different damage modes and damage signal characteristics that will eventually lead to different energy curves. Secondly, the conversion of energy is also related to the allowable deformation and it is affected by geometric factors. In the future, the discovery and methodology in this work can be used to develop fracture and failure theory for natural fiber woven FRPs. 

## Figures and Tables

**Table 1:** Basic information of bamboo strip and preform and matrix.

Reinforcement		
	Plain Weaving	Twill Weaving
Linear density of tows (tex)	3541.396	
Bulk density of bamboo strip (g/cm^3^)	0.941 (0.004)	
Areal weight (g/m^2^)	555.961 (19.601)	714.344 (17.382)
Equilibrium moisture content (%)	10.014 (0.019).	
Manufacture moisture content (%)	8.651 (0.002)	

**Table 2:** Flexural properties of bamboo textile-reinforced polymers (BTRPs).

Group	MOE (GPa)	MOR (MPa)	Total Energy (kJ)	Fracture Toughness (kJ/mm)
TW5-16	13.98 (0.09) ^b^	170.45 (7.59) ^C^	3.82 (0.41) ^bc^	0.35 (0.06) ^CD^
TW5-32	14.48 (0.64) ^b^	158.34 (3.41) ^CD^	4.85 (0.17) ^ab^	0.54 (0.06) ^B^
TW5-40	14.34 (0.18) ^b^	155.94 (5.11) ^CD^	5.69 (0.39) ^a^	0.68 (0.04) ^A^
PW5UM	11.07 (1.24) ^c^	197.18 (1.24) ^B^	5.09 (0.27) ^a^	0.43 (0.05) ^BC^
PW5UW	7.55 (0.50) ^d^	139.11 (10.01) ^D^	2.94 (0.83) ^c^	0.32 (0.09) ^D^
PW7UM S	18.73 (0.05) ^a^	217.67 (18.73) ^A^	5.23 (0.54) ^a^	0.44 (0.06) ^BC^
PW7UM F	18.78 (0.64) ^a^	230.36 (18.78) ^A^	5.00 (0.04) ^a^	0.48 (0.03) ^BCD^

Note: Analysis of variance—ANOVA of each property. Different character sets indicate significant differences (Fisher’s LSD post hoc test).

## Data Availability

The data presented in this study are available by contacting the corresponding author.
